# Mating, Sperm Transfer and Oviposition in Soft Ticks (Acari: Argasidae), a Review

**DOI:** 10.3390/pathogens12040582

**Published:** 2023-04-12

**Authors:** Julian G. Shepherd

**Affiliations:** Department of Biological Sciences, Binghamton University, Binghamton, NY 13902-6000, USA; jshepher@binghamton.edu

**Keywords:** reproduction, sperm, ticks, Argasidae, hormone, diapause, oogenesis, oviposition

## Abstract

This review addresses the physiology and behavioral events involved in the reproduction of soft ticks (family Argasidae), with special attention to the events of their adult life: mating, sperm transfer and egg-laying. Many of these aspects are held in common with hard ticks, but the repeated short duration of feeding bouts in soft ticks, in contrast to the extended single engorgements of hard ticks, has consequences peculiar to soft tick reproduction. Reviewed are the dramatic external mechanism of sperm transfer, the unusual maturation and unique morphology and motility of the spermatozoa, the mechanism of oogenesis and its hormonal control, the mystery of fertilization, the involvement of pheromones in mating, the control of reproductive arrests and the vertical transmission of symbiotes in reproduction. Jumping-off points for further investigation are discussed throughout.

## 1. Introduction

This review aims to give an analysis of the physiology and behavioral events involved in the reproduction of soft ticks (family Argasidae), with special attention to the events of their adult life: mating, sperm transfer and oviposition. While there have been excellent overviews of tick reproduction [1,2,3,4,5,6], these have often emphasized the reproduction of hard ticks (family Ixodidae), both because of their preponderance and the prevalence of the pathogens carried by them in the more developed world. Much of gametogenesis and the subsequent events during mating are largely similar in hard and soft ticks, but because the feeding behavior of soft ticks is quite different from that of hard ticks, the physiology and behavior of the oviposition of female soft ticks is also different. This difference reflects a probably old divergence of soft and hard ticks. The lack of fossil evidence for ticks older than the Cretaceous period has resulted in hypotheses for the origin of ticks as varied as the Devonian, Permian and Cretaceous periods. However, a recent extensive analysis of tick mitochondrial genomes by Mans and co-workers [7] places the origin of ticks in the mid- to late Permian, with a divergence of hard and soft ticks soon after at about the time of the Permian–Triassic boundary. A rich find of mid-Cretaceous fossils (100 Ma) supports this notion based on morphological and paleogeographical considerations [8]. Earlier work of Mans and coworkers on proteins of the salivary glands found that although the same families of proteins are represented in the saliva of both hard and soft ticks, the proteins involved in anti-hemostasis, probably the first adaptation necessary for extensive blood-feeding, are largely different in soft and hard ticks [9,10]. Apparently, the differing demands of the different modes of feeding in hard and soft ticks have also led to differences in the physiology of oviposition between the two lineages.

The most striking difference between soft and hard ticks is in their foraging strategy and mode of feeding. With a very few exceptions, soft ticks are nidicolous, i.e., they remain in burrows or nests of hosts, nearby crevices, dust pans, or similar refugia and attack their hosts when they are available in or near these refugia. Consequently, soft ticks (except most larvae) feed only for short periods (several minutes to a few hours) and then detach, often waiting for another chance to feed before molting or reproducing. Therefore, in contrast to hard ticks, many soft ticks have multiple nymphal stages. Female soft ticks usually require a blood meal before they can lay eggs (except the obligately autogenous genera, *Antricola* and *Otobius*), but the blood meal can be before or after mating. By contrast, most hard ticks in the subgroup Metastriata quest for hosts in the open environment and then travel with their hosts, though species in the subgroup Prostriata (all genus *Ixodes*) are either questers or are nidicolous. Hard ticks feed for extended periods (days), and then generally detach, molting or ovipositing at or near that location. As adults, female hard ticks must have a blood meal to lay eggs and either mate on their host (metastriate ixodids), or on or off the host (prostriate ixodids), mating being required to reach full engorgement. Hard tick females lay only one batch of eggs, whereas soft ticks usually lay multiple batches, over a longer adult lifetime. Therefore, the coordination of feeding and mating requires different stimuli in the two groups [1,4,5,6].

Given that the lore discussed below about reproduction in soft ticks is based largely on just a few of 200+ species in the family Argasidae (*Argas arboreus*, *A. persicus*, *A. walkerae*, *Ornithodoros moubata*, *O. parkeri*, *O. savignyi* and *O. tholozani*), it almost goes without saying that research on other species will undoubtedly modify and enrich the discussion below.

## 2. Morphology of Soft Tick Reproductive Systems

**Figure 1:**
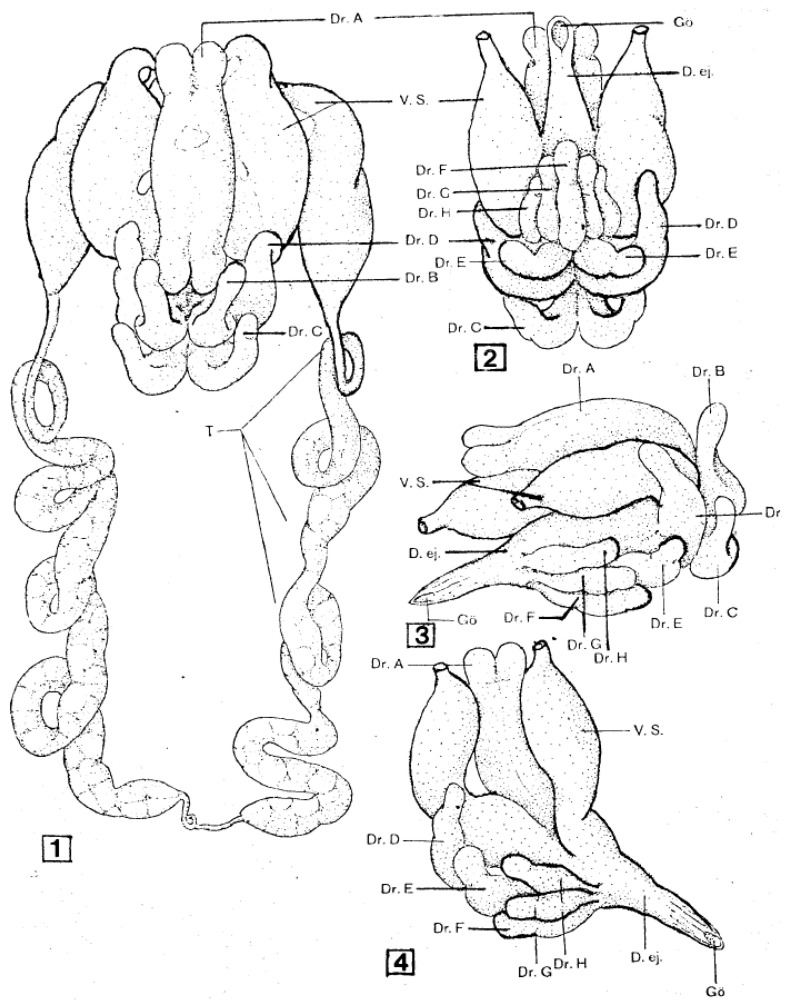
Male reproductive system of *Ornithodoros moubata.* 1. Dorsal view, 2. Ventral view of accessory glands, 3 and 4. Lateral views of accessory glands, with 4 showing the connection of the seminal vesicles to the ejaculatory duct. D.ej: ejaculatory duct; Dr. A-H: accessory glands A to H; Gö: genital opening; T: testes; V.s.: seminal vesicle. Published in *Acta Tropica*, 15, O. Wagner-Jevseenko, Fortpflanzung bei *Ornithodoros moubata.* und genitale Übertragung von *Borrelia duttoni*, 118–168. Copyright Elsevier, 1958 [13].

As shown in Figure 1, the male reproductive system is basically a loop connected to the external genital opening. The paired testes, which in most species fuse during early development, lie posteriorly and connect laterally to thin vasa deferentia that run anteriorly. These vasa enlarge at their anterior ends to form pre-mating storage organs (seminal vesicles) for mature sperm and fuse together centrally to form a large reservoir under the accessory glands. A single efferent duct runs anteriorly and opens ventrally behind the feeding apparatus. Also opening into the central reservoir of the seminal vesicles is a large and complex set of accessory glands, in soft ticks typically consisting of five (*A. persicus* [11]) or six (*A. arboreus* [12], *O. moubata* [13]) paired glands and two unpaired glands. These glands form an elaborate spermatophore outside the male’s genital opening during copulation (see below).

**Figure 2:**
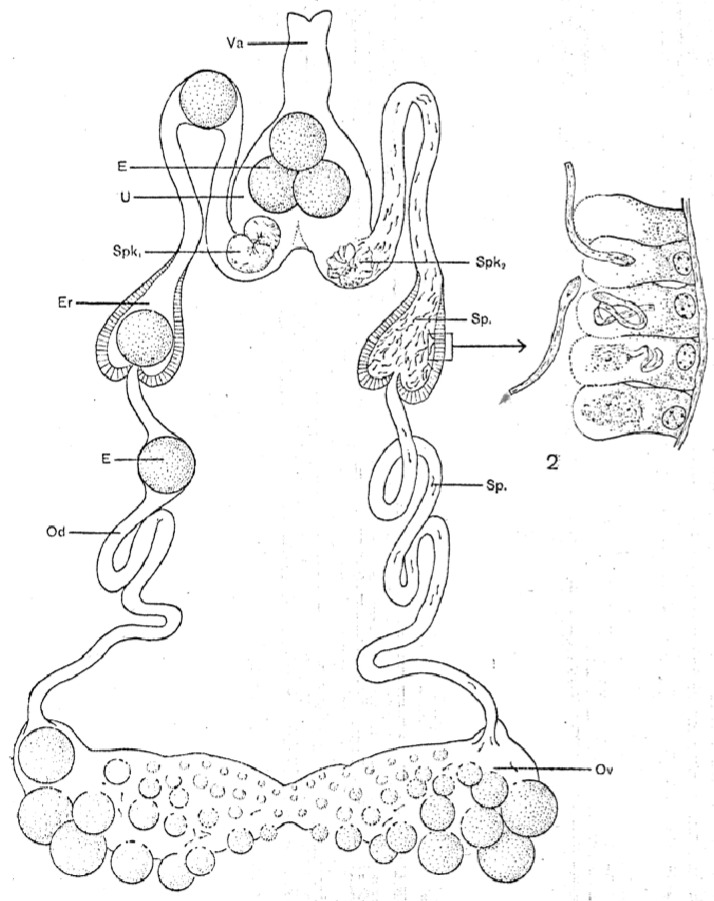
Female reproductive system of *Ornithodoros moubata*. E: egg; Er: ampulla; Ov: ovary; Od: oviduct; Sp: spermatozoa; Spk: endospermatophore; U: uterus; Va: vagina. Published in *Acta Tropica*, 15, O. Wagner-Jevseenko, Fortpflanzung bei *Ornithodoros moubata* und genitale übertragung von *Borrelia duttoni*, 118–168. Copyright Elsevier, 1958 [13].

The female reproductive system, called a “garland” by Lees and Beament [14], has basically the same organization as the male system starting with centrally fused, posterior ovaries that connect laterally to paired oviducts (Figure 2). A connecting lumen allows the passage of the eggs to the oviducts. The oviducts run anteriorly and have conspicuous enlargements (ampullae) that accumulate large numbers of sperm after mating, These unite centrally to form a median uterus (an unfortunate term as it does not perform the major functions of a mammalian uterus) in which spermatophores from one or more matings reside for up to many months or perhaps more, depending on feeding by the female. The uterus empties into an efferent vagina differentiated into a posterior cervical portion and an anterior vestibular portion. Attached at the junction of these two portions are a pair of small tubular accessory glands whose function has not been determined rigorously but seem likely to facilitate passage of the eggs during oviposition.

The organization of these reproductive structures is similar in hard ticks, with the exception that hard ticks (1) lack obvious ampullae in the female’s oviducts and (2) have lateral expansions of the vestibular part of the vagina termed lobular accessory glands [15,16]. For more details of the reproductive system morphology in soft ticks, see [1,13,16,17,18,19].

## 3. Mating Behavior and Sperm Transfer

Mating is relatively easy to observe in soft ticks as, unlike metastriate hard ticks, it takes place off the host animal. In the first detailed description of mating, Nuttall and Merriman [20] described how the male *O. moubata* crawls under the female and aligns itself face-to-face with genital openings opposite each other. After inserting all of his mouthparts in the female’s genital opening for several minutes, he pulls slightly away from the female and quickly ejaculates (less than one minute) a complex spermatophore (details below). At the same time, the male secretes copious fluid (coxal fluid: *O. moubata* [20]; saliva: *O. savignyi* [21]) that bathes the space between male and female. Several authors have suggested that this lubricates the transfer of the spermatophore, but when Robinson [22] blocked the coxal gland openings in *O. moubata*, he found successful copulation in six of eight trials. He surmised that the emission of the fluid was a byproduct of disturbance, artificial or natural, during copulation. The male then manipulates the spermatophore with his chelicerae and places the neck of the spermatophore in the female’s genital aperture. The male now waits a few minutes to leave the female, and meanwhile the spermatophore spontaneously begins to evert into the female reproductive tract (details below).

**Figure 3:**
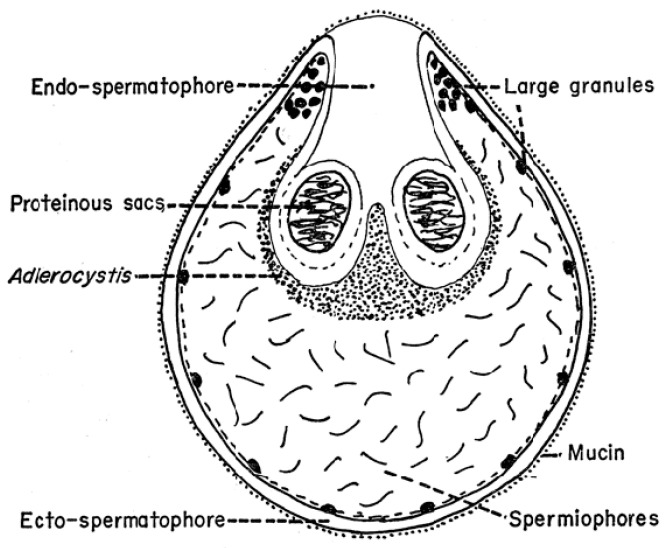
Section through a spermatophore of *Ornithodoros savignyi*. Published in *Journal of Insect Physiology*, 19, Feldman-Muhsam, B. et al., On the evacuation of sperm from the spermatophore of the tick *Ornithodoros savignyi*, 951–962. Copyright Elsevier, 1971 [24].

**Figure 4:**
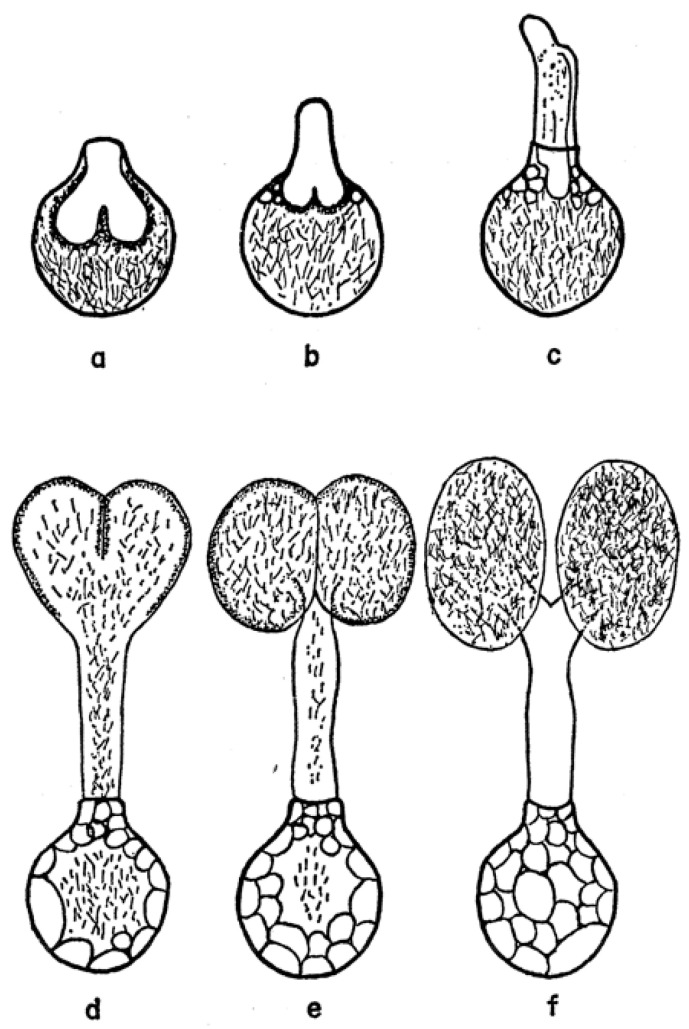
Successive stages, a to f, in the evagination of the spermatophore of *Ornithodoros savignyi*. Published in *Journal of Insect Physiology*, 19, Feldman-Muhsam, B. et al., On the evacuation of sperm from the spermatophore of the tick *Ornithodoros savignyi.* 951–962. Copyright Elsevier, 1971 [24].

As mentioned above, the structure of the newly formed spermatophore is a complex of contributions from the many different lobes of the male’s accessory gland (Figure 1). Tatchell [11] has identified the location of several of these secretions in the spermatophore of *A. persicus*, including a phenoloxidase that likely acts in tanning the outer wall of the spermatophore. While Robinson [22] first noted the extrusion of the spermatophore into the female, Feldman-Muhsam and co-workers have described the process in detail [23,24]. The outer wall of the spermatophore that will remain outside the female (thus named the ectospermatophore) is initially extruded by the male as a transparent bulb. This is then filled with sperm and seminal fluid, followed by *Adlerocystis*, a bacterial symbiont (Figure 3). Within a minute or two after placement in the female’s genital opening, the tip of the spermatophore begins to elongate and then explodes down the female’s vagina, expanding into one or two large sperm-filled bulbs in the uterus (two bulbs in *O. moubata*), these bulbs being named the endospermatophore (Figure 4). Subsequently, the ectospermatophore gradually dries and shrivels, breaking off from the endospermatophore near or past the junction of the two bulbs [25] and ultimately falling off. By the end of the evagination, the ectospermatophore is filled with bubbles and the volume of the whole spermatophore is three times its original volume. Via the careful isolation and extraction of mature ectospermatophores, Feldman-Muhsam et al. [24] showed that the carbon dioxide concentration had increased 10-fold over controls (sperm in seminal vesicles). The whole process from the male’s approach to the female and leaving her takes only about 15 min. Spermatophore formation and extrusion in hard ticks (*Dermacentor occidentalis* and *Haemaphysalis leporispalustris* [26]; eight species of Ixodidae [27]) has subsequently been shown to be largely similar, with some differing details.

## 4. Spermatozoa

**Figure 5:**
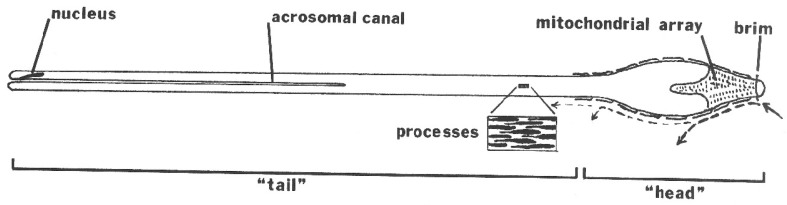
Diagram of a mature spermatozoon from within the uterus of a female *Ornithodoros moubata*. Currents along the head, as revealed by suspended particles, are indicated by the dashed lines along one side of the head. The cellular processes, which cover almost the entire surface of the spermatozoon, are shown in the blow-up and along the lateral edge of the head, but are not indicated elsewhere. The length of the entire sperm is about 450 µm. Published in *Parasitology*, 136, Resler, J.H. et al., Migration and motility of spermatozoa in the female reproductive tract of the soft tick *Ornithodoros moubata* (Acari, Argasidae), 511–521. Copyright Elsevier, 2009 [25].

**Figure 6:**
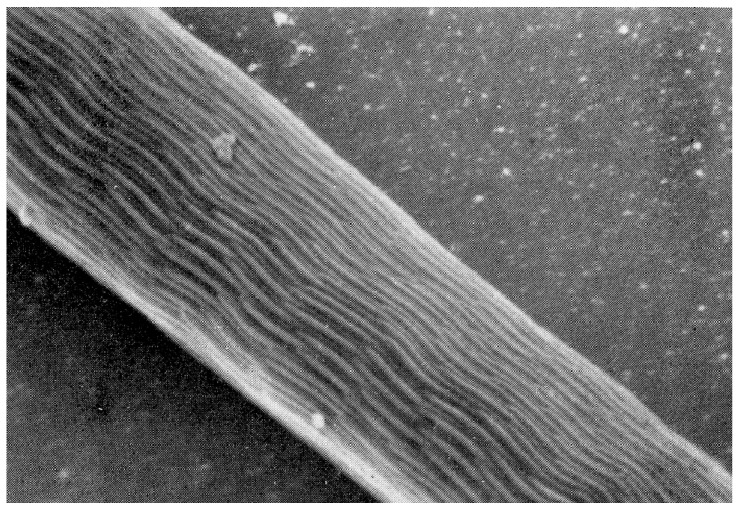
Scanning electron micrograph of an *Ornithodoros moubata* spermatozoon about halfway along its length showing the staggered array of cellular processes. Published in *Tissue & Cell*, 14, Pinkerton et al., Scanning electron microscopy of post-ejaculatory spermiogenesis in the tick *Ornithodoros moubata*, 785–797. Copyright Elsevier, 1982 [31].

**Figure 7:**
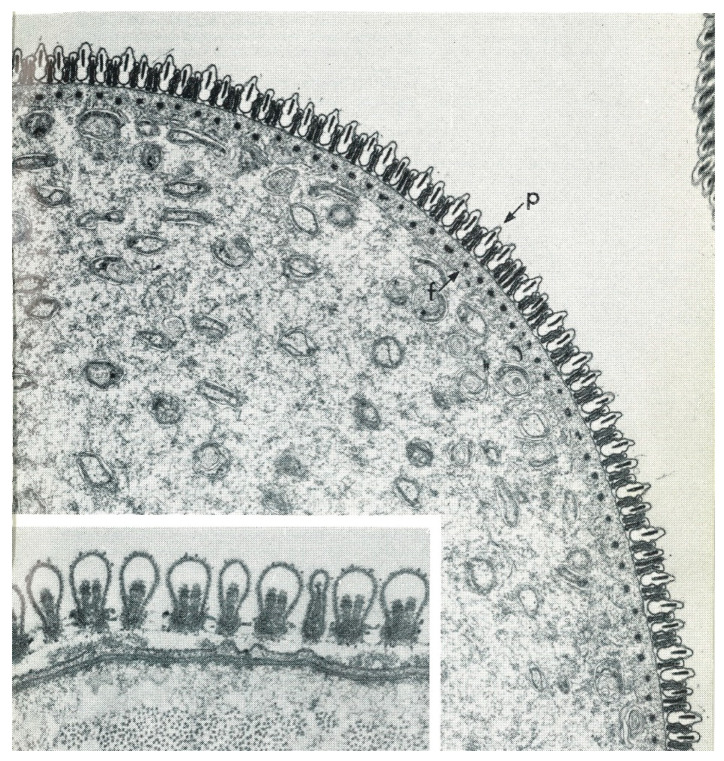
Transmission electron micrograph of a transverse section of an *Ornithodoros gurneyi* spermatozoon showing the superficial cellular processes (p) and the sub-plasmalemmal fibrils (f). Published in *Tissue & Cell*, 8, Feldman-Muhsam and Filshie, Scanning and transmission electron microscopy of the spermiophores of *Ornithodoros* ticks: an attempt to explain their motility, 413–419. Copyright Elsevier, 1976 [34].

The mature spermatozoon of ticks is extraordinary in many respects: it is very large (almost 1 mm long in *O. tholozani* [28]) with a clavate anterior end and contains a large volume of cytoplasm (Figure 5). Counterintuitively, its nucleus is located at its posterior end; the spermatozoon has no flagellum but is apparently propelled by an encircling array of longitudinal processes that result in a gliding motility (Figure 6). At its anterior end, the spermatozoon has a cap-like structure covered with mushroom-shaped projections, this cap being demarcated posteriorly by a “brim” [29], which in electron micrographs reveals a complicated substructure [30]. Behind the brim begins a staggered array of the longitudinal processes, each process about 7 µm long by 0.5 µm wide in *O. moubata* [31]. These lie adherent to the surface in living spermatozoa and cover the whole rest of the sperm surface. Each process connects narrowly with the cytoplasm at the center of the process [29] or at its anterior end [30]. A large number of mitochondria arranged in rows occupy a large part of the expanded anterior end; a single layer of mitochondria continues posteriorly under the plasma membrane of the rest of the spermatozoon. The plasmalemma of the whole spermatozoon is underlain by a relatively dense cytoplasm (Figure 7), so that living sperm in the light microscope appear often somewhat stiff, creased and opaque. Because many earlier observers surmised that this large spermatozoon (450 µm long in *O. moubata*) could not penetrate an egg in its entirety, it was initially labeled and often subsequently referred to as a “spermiophore” (=“sperm carrier”). However, inasmuch as many more conventional spermatozoa leave behind most of their extranuclear substructure on fusion with the egg, “spermiophore” seems an unnecessary term. The morphology of soft tick sperm is similar in hard ticks, with some modifications. As demonstrated in extensive electron microscopical studies of arachnid sperm by Alberti [32,33], ticks form a monophyletic clade with anactinotrichid (=parasitiform) mites within the Acari, all having what he calls “vacuolated” sperm.

## 5. Spermatogenesis

### 5.1. Precopulatory Development

Spermatogenesis begins early in larval and nymphal life by the enclosure of germinal cells in a cyst of somatic cells, as is typical in many invertebrates. The germinal cells undergo mitosis to reach a characteristic number of primary spermatocytes (16 in several species of *Argas* and *Ornithodoros*) [1]. Primary spermatocytes appear as early as the larval stage [35]. After a major growth phase, the primary spermatocytes begin meiosis and proceed into the development of spermatids (spermiogenesis). Unlike metastriate hard ticks, meiosis and spermiogenesis in soft ticks can proceed during nymphal life, producing sperm ready for transfer after the adult molt, though it usually takes a few days of adult life to reach fertility. Also, unlike hard ticks, these processes take place well into later adult life, often for many years.

**Figure 8:**
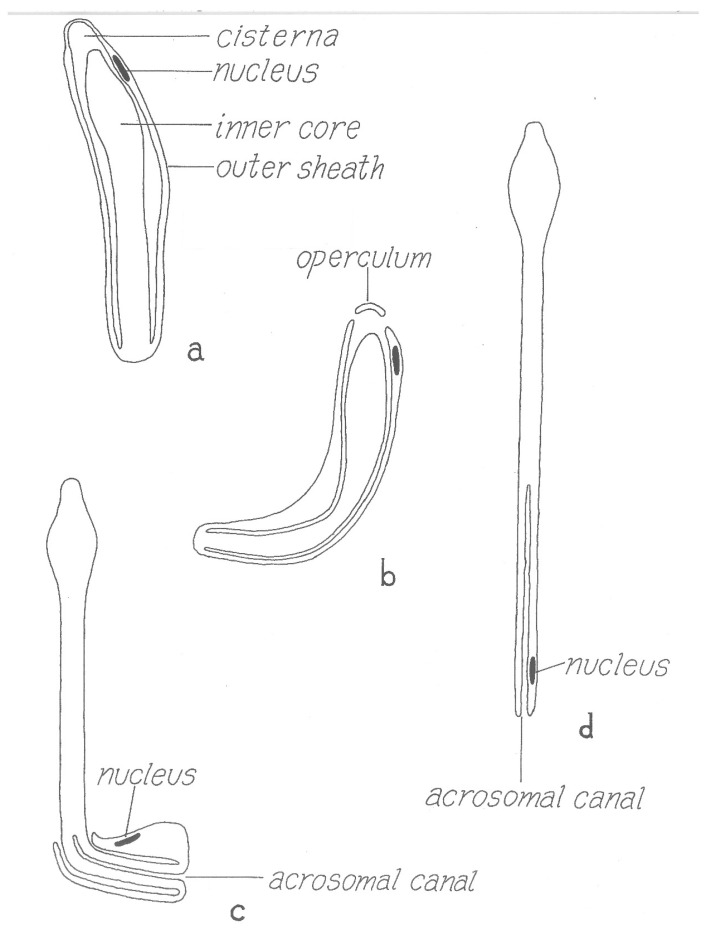
Diagram of post-copulatory metamorphosis of *Ornithodoros* sperm in the female’s uterus. (**a**), prospermium shortly after ejaculation; (**b**), rupture of the operculum induced by male accessory gland secretions a few hours after ejaculation; (**c**), elongation of the sperm accompanied by rolling back of the outer sheath and invagination of the acrosomal canal at the posterior end of the sperm; (**d**), final form of the spermatozoon showing the displacement of the nucleus to the posterior end of the spermatozoon along the acrosomal canal. Published in *International Journal of Invertebrate Reproduction*, 4, Shepherd et al., Maturation of tick spermatozoa in vitro, 311–321. Copyright Taylor and Francis, 1982 [40], www.tandfonline.com.

However, the final form of the sperm ready for transfer is very different from the mature spermatozoon described above. Electron microscopical studies [36,37,38] have clarified several aspects of this remarkably complex process that were unclear from many earlier light microscopical studies [39] (references therein). Unlike typical spermiogenesis, little cytoplasm is discarded. Instead, the cytoplasm of the initial spermatid is remodeled in several ways: (1) the nucleus is shifted to one side of the cell and joined with an acrosome formed from several Golgi apparati, (2) a series of small vesicles appear (formed either from the plasmalemma or endoplasmic reticulum) under the whole plasmalemma of the cell that eventually fuse to form a large cisterna (vacuole) inside the cell, (3) filamentous processes that project into the cisterna form within the former vesicles, (4) these processes then elongate and concentrate in one area on the inside of the cisterna and (5) this area then evaginates into the cisterna, eventually filling it. The resulting structure is thus an outer sheath surrounding an inner core (Figure 8) where the entire surface of the inner cisterna is covered with the cellular processes, except the future tip of the core. The nucleus comes to be located within the outer sheath, adjacent to the tip of the core. The product of this spermiogenesis in ticks has often been labeled simply as a “spermatid”, but products of spermiogenesis in most animals have a morphology close to the form that will unite with an egg and are thus termed spermatozoa. As the product of tick spermiogenesis is so different from its final form in the female, one of its original names, the “prospermium”, seems appropriate and descriptive.

### 5.2. Postejaculatory Development

Several thousand prospermia are transferred to the female during copulation. These immediately enter into a lengthy metamorphosis that has been called capacitation (by analogy with postcopulatory changes in mammalian sperm) or spermateleosis. Pitnick et al. [41] argue that using the term capacitation for phenomena different from the process in mammalian sperm is misleading, and so suggest the term “post-ejaculatory modifications to sperm”; a minor revision of that term seems appropriate here. The first event that occurs within a few hours after the endospermatophore arrives in the uterus is the rupture of a cap (“operculum”) from the tip of the outer sheath of the prospermium [31] (Figure 8) [42]. This event is triggered by a polypeptide originating from the male’s accessory glands that is added to the sperm upon ejaculation [40,43], and results, indirectly or directly, in a fusion of the outer plasmalemma with the cisternal plasmalemma [44]. Immediately when the operculum opens, filaments contained in the cisterna [30] are released, making the internal anatomy of the cisterna instantly visible. The function of these filaments is unknown though their rapid dissipation seems unlikely to allow a contribution to contractile function, as suggested by Feldman-Muhsam and Filshie [30]. At about the time contractions appear in the outer sheath of the prospermium, the inner core advances forward and then emerges from the hole created by the loss of the operculum (Figure 8). Films of the process show that the prospermia are often quite contorted by this process, but as most of the inner core emerges from the outer sheath, the sperm gradually straighten. Simultaneous with this elongation, the outer sheath invaginates at the posterior end of the prospermium, forming a long tube (“acrosomal canal”) that eventually extends halfway toward the anterior end of the former inner core. The nucleus of the cell, formerly at the anterior end of the outer sheath, is carried posteriorly and invaginated, ending up adjacent to the acrosomal canal close to the posterior end of the cell.

The result of this postcopulatory development (which takes about 24 h in vivo in *O. moubata* at 25 °C) is a structure that has completely turned itself inside out and doubled in length (Figure 8). Notably, all of the cellular processes that formerly lined almost all of the entire cisternal cavity are now exposed on the surface of the spermatozoon, and the former covering of the external sheath of the prospermium is now represented only in the acrosomal canal. A comparison of the surface area of the outer sheath of the prospermium relative to the resulting acrosomal canal of the spermatozoon suggests there must be a substantial loss of membrane. Terminal membranous bubbles seen in maturing spermatozoa both in vivo and in vitro, sometimes detached, appear to represent a normal mechanism for loss of membrane, though the process has not been followed in detail [31].

**Figure 9:**
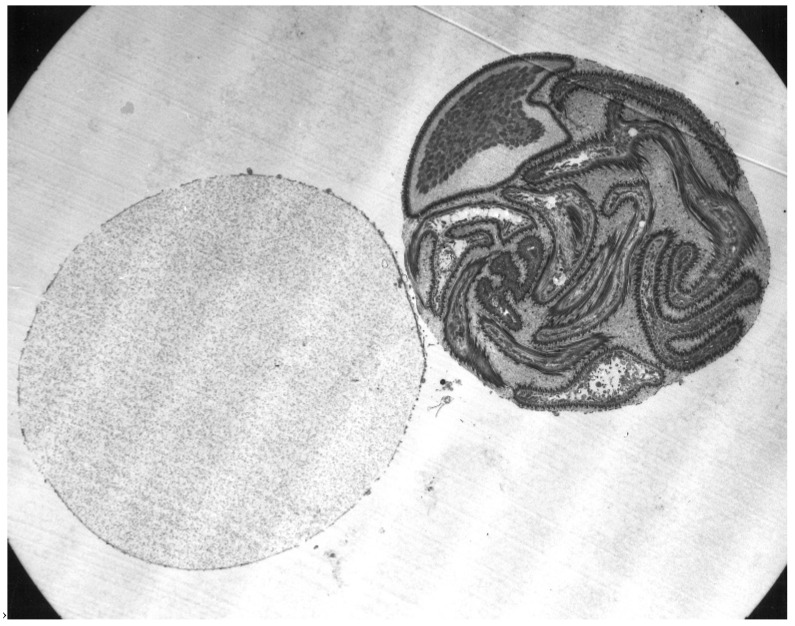
Transmission electron micrograph showing spontaneous eversion of an *Ornithodoros moubata* prospermium in vitro in the absence of accessory gland secretions. The sperm has pushed out and curled within the outer sheath without rupturing it, leaving behind the vacant-looking bulb of the outer sheath. Photo by Judy D. Hall Modafferi.

A functional reason for this extreme example of postcopulatory development is not clear, but maybe the form of the prospermium, with the cellular processes internalized, protects the processes from mechanical damage during what may be strong shear forces in the seminal fluid during the rapid formation and evacuation of the spermatophore into the female. The polypeptide that caused the rupture of the operculum apparently has little to do with the subsequent events: this was revealed by the serendipitous discovery that prospermia, left in in vitro culture for a few days without the addition of accessory gland extracts, begin to attempt elongation without the rupture of the operculum, resulting in a curious dumbbell-shaped structure (Figure 9).

## 6. Sperm Motility and Migration in the Female Reproductive Tract

Up to five different types of motility have been described [45,46], but the careful observation of motility by Resler et al. [25] indicates that only two types are actively generated by the spermatozoa, the others being passive consequences of those two types. These are: (1) gliding motility, wherein the whole spermatozoon locomotes steadily forward at a constant speed (Supplement S1), and (2) “head-curling”, seen as oscillations, contortions, or writhing movements of the anterior third of the head (Supplement S2). Sokolov (p. 158, [47]) has exemplary drawings. The gliding motility of tick spermatozoa (and other members of the anactinotrichid clade of mites) is apparently unique among arthropod sperm and perhaps among all animals as well [33]. The cellular processes described above are likely the motive organelles, but there is scant evidence for how they work. Electron micrographs in several reports show undulations of the processes [48] or of the whole sperm [34,45] and suggest that these undulations cause motility. However, these are likely artifactual as motile sperm viewed in light microscopy would show these clearly, but do not [25]. Long sub-plasmalemmal bundles of fibers that run most of the length of the spermatozoon [29] have been indicted as generators of motility [34,49]. The latter authors have identified actin by immunofluorescence in the vicinity of those fibers, but the locations of the fibers and the actin are not coincident with the most active regions of motility [25]. On the other hand, Witalinski and Dallai [49] observed a slow cessation of motility when the spermatozoa were treated with an inhibitor of actin activity, cytochalasin B, indicating an involvement of actin in gliding motility. An analogy for tick gliding motility can be found in some apicomplexan protists (first suggested by Rothschild [48]): in those organisms gliding motility involves a molecular motor consisting of an intracellular actomyosin system coupled with transmembrane proteins that adheres to the substrate [50]. Two lines of future research seem obvious: (1) an explanation of how this highly unusual motility works in ticks and (2) the origin of this type of motility, as it seems to have no relation to the motility of sperm even in the other clade of mites, the Actinotrichida (Acariformes), or in any other Arachnida [32].

Unlike gliding motility, head-curling does not obviously contribute to the movement of sperm up the female reproductive tract. This form of motility can happen in non-gliding spermatozoa, and the reverse is true: one sees gliding motility without head-curling [25]. Therefore, it appears that the two types of motility are independent of each other. One might say that head-curling looks sensory and, as noted above, the most anterior tip of the mature spermatozoon has a unique set of processes. However, beyond that, there is no clue to the function of this peculiar but quite evident head-curling motility.

The spermatozoa show the first signs of motility before the completion of their morphogenesis in the endospermatophore in the uterus [25]. However, when fully mature, the spermatozoa begin to exit the endospermatophore through its ruptured connections with the ectospermatophore (see above) and ascend the female oviducts. In spite of previous assertions in the literature (without strong evidence) that the spermatozoa are transported by the action of the oviducal musculature, Resler et al. [25] described several lines of evidence indicating that the spermatozoa of *O. moubata* move up the oviducts under their own power, and observed no indication of assistance from the muscles in the walls of the oviducts. They did show experimentally that this movement occurred only if the female had had a blood meal, supporting previous assertions to this effect [51,52]. The spermatozoa accumulate in large numbers in the expansions of the oviducts, the ampullae (as shown in Figure 2), but then, despite some former contentions otherwise, some sperm continue moving up into the ovaries [12,13,14]. In unfed females, mature spermatozoa remain in the endospermatophore in the uterus [25,47]. Unfed females evidently retain these sperm in a viable condition for many months and even years (*O. savignyi*, 11 months, [52]; *Ornithodoros papillipes* and *Alveonasus lahorensis*, 4 years, [35]; *A. arboreus*, 10 months, [53]; *Argas (Ogadenus) brumpti*, 4 years, [54]). Whether the trigger for movement after a blood meal comes directly from some factor in the blood meal or more indirectly from female tissues seems well worth further investigation.

A final step in the maturation of the spermatozoa occurs only after the sperm are in the oviducts for a few days (*O. parkeri*) [55]. The acrosomal canal at the posterior end of the spermatozoon partially everts (average 28 ± 8 µm in *O. parkeri*) exposing the nucleus on its surface [30,31]. This would seem to bring the nucleus into a propitious location for fertilization (syngamy), which will be discussed below.

Another striking phenomenon seen in both hard and soft ticks is the penetration of spermatozoa into the tissue lining the oviducts (see Figure 2). Sokolov [47] describes in detail how in both *O. papillipes* and *Dermacentor marginatus* the spermatozoa curve toward the oviduct wall, stand head-first perpendicular to the wall attaching to it, and then penetrate not only the tissue but actually into the oviducal cells, often curled into a tight ball. In *Dermacentor andersoni*, the spermatozoa penetrate all the way to the basement membrane of the oviducal tissue [56]. At later times, only fragments of the sperm remain, apparently as a result of phagocytic digestion [47]. The oviducal tissue is considerably destroyed in the process.

## 7. Oogenesis

**Figure 10:**
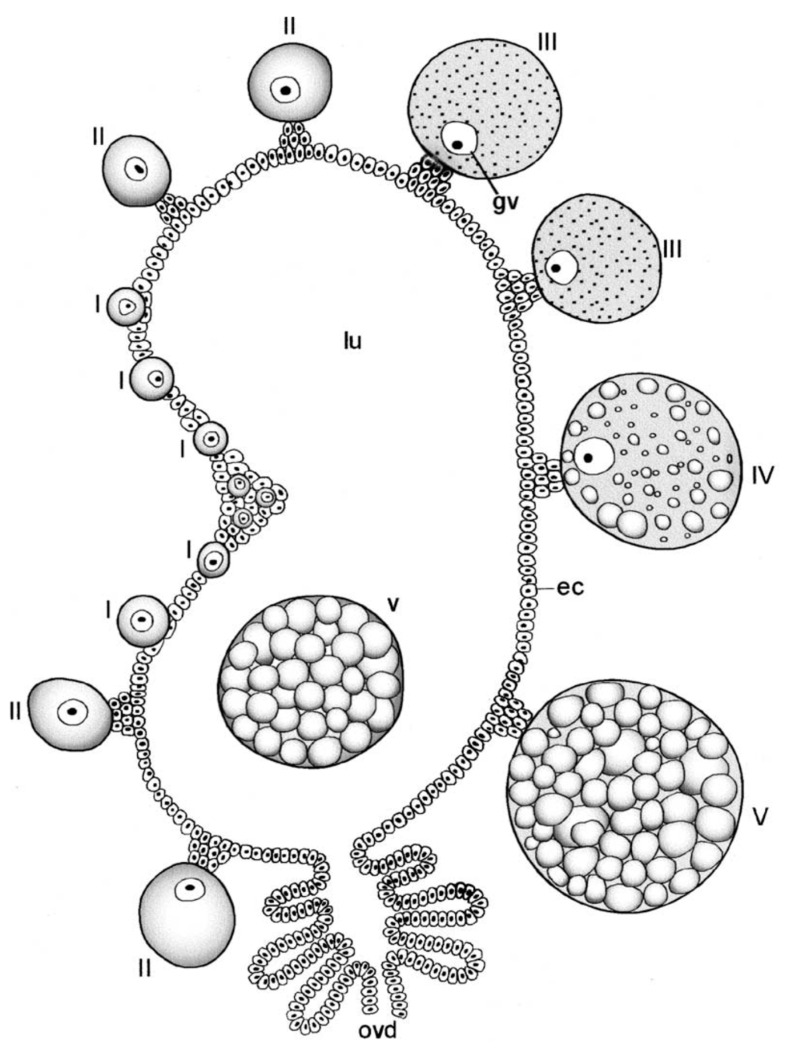
Stylized diagram of oocyte development within the ovary of *Rhipicephalus microplus*. I–V, Balashov stages of oocyte development (see text); ec, ovary epithelium; gv, germinal vesicle (nucleus); l, lumen of ovary; ovd, beginning of oviduct; v, ovulated oocyte. Similar development occurs in soft ticks. Published in *Veterinary Parasitology*, 129, Saito et al., Morphological, histological and ultrastructural studies of the ovary of the cattle tick *Boophilus microplus* (Canestrini, 1887) (Acari: Ixodidae), 299–311. Copyright Elsevier, 2005 [60].

Oogenesis has been described in detail by Balashov [1] and Diehl et al. [57], so only a brief description will be given here. Oogonia are initially embedded in the single- or few-celled epithelial layer of the ovary supported exteriorly by a thin but resilient layer of connective tissue (tunica propria) (Figure 10). After mitoses, the oogonia become primary oocytes in what Balashov defined as stage one (I). Their cytoplasm and nuclei then grow dramatically, causing the oocyte to expand out beyond the tunica but remaining connected via a stalk formed of epithelial cells, the pedicel or funicle (Balashov stage II). The oocytes then begin to accumulate yolk and consequently enlarge, and an eggshell begins to form (Balahov stage III). Balashov stage IV is marked by the end of vitellogenesis, the completion of egg-shell growth and a thinning of the pedicel. At Balashov stage V, the mature eggs are ovulated through the pedicel into the ovarian lumen and move down to the oviducts. Contrary to some suppositions, the eggshell is completely proteinaceous and not chitinous [14]. Ovulation has never been observed and its stimulus and mechanism are unknown. Detailed studies of soft tick oogenesis have been undertaken in *O. moubata* by Wagner-Jevseenko [13], Diehl [58] and Diehl et al. [57] and in *A. arboreus* by El Shoura et al. [59].

One notable feature of the developing oocytes is their lack of association with an adjacent follicular epithelium or nurse cells, a characteristic relationship in most arthropod ovaries that functions to provide organelles and/or substances that provision the oocytes. In a preliminary report, Aeschlimann and Hecker [61] showed extensive interdigitations between the membranes of pedicel cells and oocyte cells in *O. moubata* and proposed a supportive association. However, in a detailed ultrastructural study of oogenesis in *D. andersoni*, Brinton and Oliver [62] argued against this interpretation in favor of a simple adhesive function between oocyte and pedicel. That does mean that the oocyte generates its own structures and acquires all nutrients from outside the ovaries. Both of the foregoing pairs of authors and others [14] agree that, unlike many other arthropods, the oocyte makes its own shell.

With the few exceptions mentioned previously, female ticks need to take a blood meal and mate in order to lay a clutch of eggs. Female metastriate ticks need to both find a host and then mate on the host in order to develop and lay a once-only batch of eggs. While this is clearly an evolutionarily successful strategy, so also is the argasid strategy, whereby argasids can mate or engorge in either order, often at long intervals, and can do this many times laying many clutches of eggs. In compensation, their blood meals are smaller and more intermittent than those of hard ticks and result in producing much smaller batches of eggs (typically hundreds) than do hard ticks (thousands). Some females of some species of soft ticks (e.g., *A. arboreus* [12] can, after mating, lay a clutch soon after their nymphal-adult molt, sponsored by blood remaining in their midgut from a nymphal meal. However, usually an adult female engorges and then when she mates, she oviposits a clutch; typically, she will repeat this several times in her lifetime in what are termed gonotrophic cycles. However, when Aeschlimann and Grandjean [63] tested female *O. moubata* that were fed regularly but mated only once, the females could lay up to eight clutches, but clutch size decreased in later clutches, sustained by the retention of some blood in the midgut after each clutch [57]. Conversely, if a female *O. moubata* is fed once without mating, she will begin to develop some eggs through Balashov stage II or III and a very few further, but those will then begin to be resorbed until she mates, and then when she mates (up to 5 months later) she will then develop and lay a clutch [64].

These results indicate that egg maturation and oviposition are dependent on two stimuli, one from mating and another from feeding. Reasoning that elements of the spermatophore might induce egg development, Aeschlimann [64] injected variously homogenates of whole spermatophores, male seminal vesicles, male accessory glands, ovarian tissue, or the intact spermatophore sperm of *O. moubata* into the hemocoel of virgin, fed females; he found that the spermatophore, seminal vesicles, prospermia effectively induced oviposition, but that male glands and ovarian tissue did not, indicating that sperm contained a stimulus for oviposition. Further, if uterine spermatozoa were incubated in a physiological medium for 20 h, and then this medium was injected into virgin, fed females, the medium induced 94% oviposition [65]. Subsequent experiments [44] assaying medium of incubated sperm (as above) from spermatophores at different times after mating (0–48 h) showed that fresh prospermia had not released any factor stimulating vitellogenesis and/or oviposition (now termed vitellogenesis-inducing factor–VIF). However, as prospermia underwent postcopulatory development, VIF was released sometime between 0 and 12 h after spermatophore formation. Molecular sieve analysis and the measurement of temperature sensitivity indicated that VIF is a protein of 150,000 daltons or larger. By means of electrophoresis of the incubation media of sperm from differently aged spermatophores, Sahli et al. [44] identified two proteins that were maximally present 12 h after spermatophore formation, though apparently these were not extracted and assayed for VIF activity. Thus, since VIF was also found in homogenates of male seminal vesicles, VIF is already present in prospermia before ejaculation and then released during postcopulatory development, perhaps when the internal cisterna opens and releases its vesicles [66]. These findings in *O. moubata* seem likely to apply to other *Ornithodoros*, as the injection of the sperm of *O. tholozani* and *O. tartakowski* into virgin female *O. moubata* induced oviposition [65]. All of the above experiments identify VIF by injection into the hemocoel of virgin hosts; a desirable sequel would be to artificially inseminate virgin females (apparently technically feasible: [67]) and see if VIF will exert its effects as it does in vivo.

To see if VIF might act via the neurosecretory action of the synganglion, Aeschlimann [63] injected extracts of the synganglia of fed mated females into fed virgin females. These induced about 40% of subjects to oviposit, whereas extracts from virgin, fed females resulted in only 8% ovipositing. However, when repeating these experiments, Connat et al. [68] found that the implantation of synganglia from virgin females, mated females and males all equally induced some vitellogenesis but not oviposition, indicating a non-specific effect of implantation.

Thus, while a chemical stimulus for yolk formation (vitellogenesis) upon mating is well-established in *O. moubata*, it apparently is not the only stimulus. The implantation of metal beads into the vagina or uterus of fed virgin females induced vitellogenesis and oviposition to a level comparable to mated females, though the number of eggs laid was significantly lower (Ducommun 1984, quoted in Connat et al. [68]). Presumably, these beads mimic the effects of male mouthparts or more likely mechanical stimulation by spermatophores. As with many developmental events, the induction of vitellogenesis and oviposition in these ticks is the result of more than one at least partially redundant stimuli.

Similar experiments with other argasids have revealed both similarities and differences. Leahy and Galun [69] placed plastic beads in the vagina or injected homogenized male accessory glands + seminal vesicles into virgin female *A. persicus* and concluded that both increased the number of mature eggs. However, these results lacked statistical verification and/or appropriate controls. Working with *A. arboreus*, Khalil and Shanbaky [70] found that injections of homogenized testes or male accessory glands injected into the hemocoel of virgin females (as Aeschlimann et al., cited above) significantly increased vitellogenesis to ovulation at levels equivalent to those in normally mated females, but only a few eggs reached the uterus and none were laid. The placement of silica beads in the vagina or uterus had no stimulatory effect. Thus, unlike the case in *O. moubata*, accessory glands can stimulate vitellogenesis, but as in *O. moubata*, sperm could stimulate vitellogenesis, though the maturity of the sperm was not equivalent to those tested by Aeschlimann et al., so comparison is problematical.

## 8. Hormonal Control of Egg Development

The hormonal control of oogenesis has been a strong focus in research on tick reproduction, and excellent recent reviews of the field are those by Rees [3,4], Roe et al. [71], Ogihara and Taylor [19], Ogihara et al. [72]. The focus has been heavily on hard ticks, and as female soft ticks have reproductive cycles different from those of hard (especially metastriate) ticks, this review will concentrate on the reproductive physiology of female soft ticks.

The results of the experiments cited in the preceding section indicate several likely control points in the production of eggs: (1) the initiation of oocyte maturation–Balashov stage I to II, (2) the initiation of vitellogenesis–Balashov stage II to III, (3) ovulation–Balashov stage IV to V, (4) oviposition. Although the observation and experiments cited above indicate that egg production can halt at steps 3 and 4, nothing seems to be known about their specific triggers, so this review will address steps 1 and 2. However, it should be pointed out that much work has been undertaken on the initiation of yolk production, but less on the transition of oocytes from stage II to stage III, i.e., the induction of yolk uptake by oocytes. These two stages seem to follow sequentially, so might be controlled by the same hormonal stimulus, but this needs further elucidation. An interesting phenomenon specific for soft ticks is how stage I, initiated by a blood meal, only activates a fraction of oocytes, enabling multiple subsequent gonotrophic cycles. On the other hand, such selective activation occurs in gametogenesis in many other organisms, e.g., oogenesis in mammals.

Taking an example from insect hormonal studies, Shanbaky and Khalil separated the synganglion from the ovaries of the mated female *A. arboreus* [73] or *Argas hermanni* [74] by ligation behind the second pair of coxae. Ligation one hour after feeding resulted in oocyte maturation no different from unfed females. Ligation on sequential days after feeding resulted in an increasing number of developing eggs, reaching a maximum on Day 4, when those females had developed eggs equivalent to unligated fed females. Another group of mated females were fed and injected with an extract of synganglia removed from other females 3 days after feeding, i.e., “active” synganglia, and immediately ligated. These females developed eggs to a level comparable to normal unligated fed females. However, when injected with extracts of synganglia from females one hour after feeding (“inactive” synganglia), the ovaries in host females showed a lack of development equivalent to uninjected controls. It should be noted that there was no mention of a statistical test of the difference between the two experimental groups. Nevertheless, these results indicate the release of a gonadotropin from the synganglion that induces egg development over several days after a blood meal, although the blood meal apparently has other effects essential for egg development as neither active nor inactive synganglia would stimulate the ovaries of unfed females. The protein analysis of the hemolymph and ovaries showed that the action of the synganglion coupled with a blood meal result in the release of vitellogenins into the hemolymph and incorporation into the ovaries. However, Oliver et al. [75] found that transplanting whole synganglia from even virgin unfed females of *O. parkeri* into the posterior portions of mated females ligated two days after feeding stimulated vitellogenesis, in contrast to the results cited for *A. arboreus*. They postulated that this was due either to a species difference or that the implantation of whole synganglia, in contrast to synganglial extracts, might have allowed the activation of synganglia to release a vitellogenic stimulus.

Via biochemical, electron microscopical and immunohistochemical analysis, Chinzei and Yano [76] presented convincing evidence that the major source of vitellogenins in female *O. moubata* is the fat body. Then, by ligating mated females soon after feeding as did Shanbaky and Khalil, Chinzei and Taylor [77] established the same role for the synganglion in *O. moubata*, calling the synganglial gonadotropin “vitellogenesis-inducing factor” (VIF: somewhat unfortunate as Sahli et al. [44] used this same term for the pheromone that developing sperm secrete after ejaculation into the female–detailed in the section above). Vitellogenins appeared in the posterior parts of ligated ticks soon after ligation, but not in anterior parts in spite of the presence of fat body there. However, if unligated three days after feeding, vitellogenins appeared there. On this basis, they postulated that VIF did not induce vitellogenin release directly, but rather induced a second factor that they called fat-body stimulating factor, FSF.

K. Ogihara, M. Ogihara, Taylor and associates have identified FSF as ecdysteroids produced in the ovaries of mated fed *O. moubata* that stimulate the fat body production of vitellogenins and also perhaps regulate oocyte development and oviposition. Ogihara et al. [72,78] showed that ecdysteroid titres rise sharply after engorgement in the mated female *O. moubata*, and this is followed a few days later by a sharp rise in hemolymph vitellogenins. As mentioned earlier, virgin females show the development of oocytes after engorgement, but not to late stages of development or oviposition. Ecdysteroid titres rise a little in these females followed by vitellogenin synthesis, but the injection of exogenous ecdysone will induce development to oviposition, though not quite to the levels shown by mated females. On this basis and the evidence of the early production of vitellogenins in the midgut as well as fat body, Horigane et al. [79] proposed a parallel pathway model: (a) engorgement without mating induces early vitellogenin production by the midgut and fat body but does not result in oviposition and (b) engorgement with mating induces much more vitellogenin production mainly in the fat body followed by oviposition. They noted earlier that, because of the potential for the dissociation of mating and engorgement in soft tick gonotrophic cycles, soft ticks provide a better system than do metastriate hard ticks for studying these pathways. Ogihara et al. [80] extended these studies with the identification of two genes in the 20-hydroxyecdysone (20E) synthesis pathway, *spook* and *shade*. The former is involved in the earlier steps of the pathway, and the latter in the final step, the hydroxylation of ecdysone to the effective hormone 20E. *spook* is specifically expressed in the ovaries, *shade* more widely, including the midgut. The same group has recently identified the target-of-rapamycin (TOR) pathway as essential for vitellogenesis in *O. moubata* [81], as previously shown in hard ticks and blood-feeding insects. The injection of rapamycin significantly delayed vitellogenin secretion and ovarian development in both virgin and mated females, though both secretion and development recovered later, presumably because of the metabolic degradation of the rapamycin. Both phenomena were much lower in virgin than in mated females, correlated with the lower 20E production in virgin females. This lower level of vitellogenin apparently results in the incomplete development of oocytes and consequent lack of oviposition. The full elucidation of these pathways is promised and may also lead to an elucidation of how secretion by the synganglion functions in initiating egg development.

By analogy with most insect species, it had long been thought that juvenile hormone (JH) might be involved in female reproduction in ticks. This idea came initially from experiments applying JH inhibitors [82], but several later studies could not show the effects or presence of JH [83,84,85]. More recent studies have shown that parts of the metabolic pathway leading to JH are present in hard ticks [86], so this pathway may yet be shown to have some role in tick reproduction.

In summary, a pheromone is released from developing spermatozoa after ejaculation, which directly or indirectly triggers neurosecretion by the female’s synganglion. The synganglion then releases neurosecretion that induces ecdysteroid synthesis by the ovaries and probably other tissues, and then ecdysteroids induce vitellogenesis by the fat body and midgut. This egg development appears to result from the interaction of two closely related pathways, the TOR-signaling and the ecdysteroid synthetic pathways. This scenario is mostly based on experiments in *O. moubata*, and it seems likely that modifications will emerge from the study of other argasid species. The nature of the neurosecretions is an open question, though transcriptomes of the synganglia of the adult hard tick *Dermacentor variabilis* show a number of transcripts for signaling peptides potentially relevant to reproduction [87,88].

## 9. Fertilization, Egg Passage and Oviposition

Fertilization, in the sense of the union of male and female gametes, has been shrouded in mystery and controversy. To this date, it is unknown exactly how or where it occurs, and whether the whole or a part of the sperm penetrate the eggs. Several old studies offer tempting tidbits: Christophers [89] reported seeing fragments of sperm in ovarian eggs of *O. savignyi*, and Samson [50] reported she was able after many attempts to artificially inseminate an egg of *Ixodes ricinus*, though she did not track subsequent embryonic development. Some extensive studies [13], Goroshchenko–cited by Balashov [1], Garcia-Fernández et al. [90] have favored the oviducts as the locus of fertilization, citing the abundance of sperm in the ampullae of the oviducts and their apparently active penetration into oviducal cells. However, several careful reports in *O. moubata* of the synthesis of an apparently complete eggshell formed by the oocyte in the ovary have made it difficult to imagine the subsequent penetration of the egg in the oviducts [14,58]. Several workers have detected either a thinning or depression of the eggshell opposite the pedicel (*O. moubata* [58]; *A. arboreus* [59]), or a 7 µm opening that extends through the eggshell directly in line with a channel between the pedicel cells that extends to the lumen of the ovary (*D. variabilis* [62] (Figure 33). Khalil reports seeing sperm within the oocyte cytoplasm in the ovary of *A. arboreus* [12]: her photo does not show this clearly, though her other pictures of what may be male pronuclei in ovarian oocytes are more convincing [12] (Figure 49). Somewhat corroborating this, Garcia-Fernández et al. [90] show light micrographs of Balashov stage-III oocytes containing two structures that stain with specific DNA stains (Feulgen and DAPI). Several significant attempts in my laboratory using different techniques have also failed to clarify the issue. Finding the site and mechanism of fertilization has been a graveyard of effort, but it seems likely that tick oocytes are penetrated before shell formation (early Balashov stage III or earlier) by only the nucleus located at the tail end of the spermatozoon. Perhaps a carefully timed electron microscopical study would shed some light.

Ovulated eggs pass into and continue down the oviducts apparently by dint of the musculature in the tunica propria that lines the ovaries and oviducts. *O. moubata* eggs are initially soft and thin-walled but considerably harden after they pass from the ampullae into the uterus, perhaps via secretions of the uterus [58]. Eggs are often retained in the uterus for considerable periods as oviposition often occurs in bouts with intervening periods without oviposition.

During oviposition, the vestibular portion of the vagina prolapses forming an “ovipositor” as each egg is extruded. Tubular accessory glands open at the junction of the cervical and vestibular parts of the vagina, and inasmuch as they are clearly secretory, it seems likely that they add some lubricant and perhaps initial waterproofing, but the function of these glands has apparently never been elucidated.

**Figure 11:**
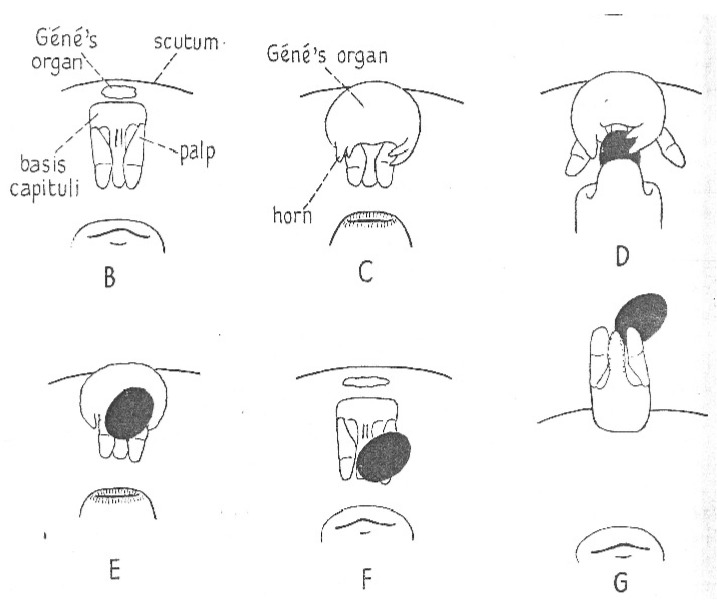
B to G: sequential movements of Gené’s organ in Ixodes ricinus. Similar in all respects to the organ of *O. moubata* except for image G: the egg is not thrown but just dropped in *O. moubata*. Published in *Quarterly Journal of Microscopical Science*, 89, Lees and Beament, 1948, An egg-waxing organ in ticks, 291–332. Reproduced with permission of Cambridge Univ. Press [14].

As the egg emerges from the ovipositor, a remarkably bizarre organ, Gené’s organ, everts from the region anterior of the mouthparts. First described by Gené almost two centuries ago, the behavior of the organ and details of its structure were described by Robinson and Davidson [17]. However, the function of the organ was not elaborated until a thorough study of the organs of *O. moubata* and *Ixodes ricinus* was published by Lees and Beament [14]. As the mouthparts are depressed, the organ emerges from a narrow slit, everting into a rather large pouch with arms that grab the egg proffered by the ovipositor (Figure 11). While the gland pulsates, the female rotates the egg for a few minutes, coating it with a copious secretion, and then releases the egg, which is added to a heap of already treated eggs under the female. The organ is then retracted internally, and the cycle is repeated when the next egg is produced. Although not all of the egg contacts the gland, the secretion spreads readily over the whole surface of the egg. Lees and Beament showed that the coating is a wax produced by the organ; without the wax, the eggs quickly shrivel and fail to develop. They successively blocked then unblocked the opening of the organ; eggs laid when the organ was blocked shriveled and died, but those laid when unblocked hatched normally. They also elaborated on the mechanics of the process and chemical nature of the waxy secretion and its precursor that accumulates in a large space between the epidermis and cuticle of the gland. A study of oviposition by Edelmann and Gothe [91], accompanied by details of timing and elegant photographs, reported the same process in *A. walkerae*. Since the wax secreted by Gené’s organ is crucial for egg survival, this phenomenon represents a critical vulnerable point in the life cycle of soft ticks.

## 10. Mating Capacity of Males and Fecundity of Females

Males have a prodigious capacity for mating and producing spermatophores. Feldman-Muhsam [92] found that single males of *O. savignyi* could mate up to thirty-two times even if unfed, and single males of *O. tholozani* could mate up to thirty-three times, though in that maximal case, the last seven matings were infertile. Jobling [93] reported several instances of *O. moubata* males mating 10 times. Although perhaps the product of multiple males, Lees and Beament found female *O. moubata* with up to 12 endospermatophores in their uteri [14]. Although not tested rigorously, it appears that female *O. tholozani* show no inhibition of receptivity to sequential matings [92], but this seems worth further investigation.

The fecundity of soft ticks varies with the number of blood meals and matings, and of course among different species. In *O. moubata*, one blood meal followed by one mating resulted in a lifetime average production of 244 eggs; egg production from a single blood meal followed by multiple matings was not significantly different [63]. However, multiple blood meals and multiple matings at intervals would produce much more (not quantified–ibid). The recorded lifetime fecundities of soft ticks in natural or laboratory conditions are sparse, and these either result from or are likely to result from laboratory experiments. Jobling [93] recorded an average (n = 9) of 877 eggs per female laid in six batches in a 29–30 °C laboratory colony of *O. moubata*. Pospelova-Shtrom (p. 77, [94]) lists averages (with no information on environmental conditions) for *O. moubata*: 1217, *O. coriaceous*: 1158 and *O. erraticus*: 1157. In lab experiments at 28 °C, Khalil [95] records 66–81 eggs produced by *A. arboreus* over 32 days after a blood meal, though it is not clear if this period would be the end of oviposition.

## 11. Pheromonal Involvement in Reproduction

While the involvement of pheromones in the reproduction of hard ticks has been the subject of much attention and progress, particularly in view of its potential for tick control, understanding of the seemingly likely involvement of pheromones in soft tick reproduction is minimal. In hard ticks, especially metastriate ticks, three pheromonal steps in the interaction between male and female have been identified: (1) an attractant, dichlorophenol, (2) a mounting pheromone and (3) a genital pheromone. Sonenshine [96] and Mulenga [97] give detailed reviews of these pheromones and their application to control in hard ticks.

Although there is little known about true sex pheromones in soft ticks, the existence of general arrestment pheromones (formerly known as assembly pheromones) was first discovered and investigated in soft ticks [98], and they have subsequently been shown to exist in most hard ticks. These arrestment pheromones cause the aggregation of ticks of both sexes and apparently do not function mainly for mating purposes, though they seem likely to facilitate it. Leahy et al. devised a simple assay for these pheromones by dividing a circular piece of filter paper in a petri dish into equal sectors. Then, the impregnation of one sector with colony exudate of *Ornithodoros porcinus* (a sibling species within the *O. moubata* complex–Walton [99]) resulted in strong aggregations of both sexes, nymphs and larvae of *O. porcinus* as well as congeneric species and even confamilial species (*A. persicus*, *A. brumpti*) [100,101]. Aggregations were sufficiently tight that the whole mass could be picked up as a unit! Using a similar choice arena for *A. walkerae*, Gothe and Kraiss [102,103] also identified an arrestment pheromone in that species. By blocking the anus, genital opening and/or coxal glands in various combinations, they identified the anus as the principal source of pheromone. This pheromone extracted from *O. porcinus* was subsequently found to attract *A. persicus* and other tick species, including some hard tick species, and was identified as guanine, the principal excretory product of ticks [104]. Further, as an arrestant for *A. persicus*, Dushábek et al. [105] showed that a mixture of several purines was superior to guanine alone, though this was not validated by a statistical test.

Neitz and Gothe [106] also identified a volatile arrestant for male *A. walkerae* by trapping the volatile effluent of 300 engorged virgin female A. walkerae in either hexane or by freezing at liquid nitrogen temperature and subsequent extraction with diethyl ether. Testing the aggregation potential of both extracts, both showed a strong attraction for engorged male ticks, although no control tests were performed with male effluents or female respondents. Through again collecting the effluent of engorged females and then blocking the anus, genital opening and/or coxal glands in combinations, the origin of this volatile pheromone was identified as the genital opening, though this conclusion is somewhat vitiated by the lack of statistical tests and replication [107].

Apparently, the only proven sex-specific pheromone that has been demonstrated for argasids comes from experiments by Schlein and Gunders [108] using *Ornithodoros erraticus*. Coxal fluid collected from ticks was applied to fed nymphs, and then when a male, after mounting a nymph, crawled under and aligned himself with the nymph as he would a female, that was deemed a mating attempt, as defined by Nuttall and Merriman [20]. Coxal fluid from nymphs or freshly molted females applied to nymphs elicited very few mating attempts, as did uncoated nymphs. Coxal fluid from females previously exposed to males (probably mated) and applied to nymphs induced significantly more mating attempts, though not as many as when untreated females were used as subjects instead of nymphs. Interestingly, leg-amputated or immobilized females, or rubber decoys coated with female coxal fluid, elicited no response, apparently because female mobility is necessary for the male mating response. The authors noted the need to test coxal fluid from males and other life-stages more rigorously. Similar tests with O. savignyi by Mohamed et al. [109] supported the specific efficacy of female coxal fluid as an attractant for males. They showed further that males orient to females up to 9 cm away, and when they climb onto females, they palpate the female’s coxal gland openings, and show less interest if coxal glands are sealed.

## 12. Reproductive Diapause

A cessation of reproductive activity in unfavorable environmental conditions is widespread among ticks [110] (Table 13.4). The commonest adverse environmental condition is adverse weather that limits food supplies, and so is prevalent in temperate environments, particularly among hard tick species that are more abundant than soft ticks at higher latitudes where seasonal conditions are more variable. However, both temperate and subtropical soft ticks have reproductive diapauses, probably largely caused by lapses in host availability. Such reproductive diapauses are manifested by a delay in oviposition even if females have mated. The normal delay between mating and oviposition, the “pre-oviposition period”, is normally a matter of less than a few weeks, depending on temperature. However, a diapause is signaled if that interval extends to more than a month and, as it is usually seasonal, it often extends to a large part of a year (e.g., 4–7 months in *Ornithodoros kelleyi* [111], 10–11 months in *Argas reflexus* [112]).

The only in-depth investigation of a determinant for reproductive diapause in soft ticks has been the work of Khalil [113,114] on a population of the bird tick *A. arboreus* near Cairo, Egypt. This population lives around cattle egret (*Bubulcus ibis*) breeding colonies; the ticks feed and reproduce only when their migratory hosts are in residence during late spring and summer (April to September) [115]. If mating happens toward the end of those months, oviposition is delayed until the next nesting season of the host, about 8–9 months [53]. Ticks were collected in mid-summer (a natural long photoperiod) and then exposed in the laboratory to a short photoperiod (LD 9:15). Over the next ten weeks, progressively more of the ticks entered diapause until, by ten weeks, over 90% were in reproductive diapause, as measured by a delay in oviposition greater than 40 days after mating (non-diapause delay averages 7–17 days [113]). If females in reproductive diapause were then placed in a long photoperiod (LD 16:8), diapause was terminated in close to 100% of females by 10 weeks of exposure [114]. Groups of ticks held at various constant temperatures had no effect on diapause incidence. These experiments indicate photoperiod as the principal determinant of diapause in this population. However, seasonality in some argasid species is apparently not cued by photoperiod, as their hosts live in caves or other dark environments, e.g., *O. kelleyi* that parasitizes cave-dwelling bats [111].

Building on their finding of a gonadotropin from the synganglion that stimulates oviposition (see above), Khalil and Shanbaky [116] showed that the injection of synganglial extracts from summer-collected females would terminate diapause in 50% of mid-diapause (October) ticks and 100% of late diapause (January) ticks. On the other hand, the injection of synganglial extracts from diapausing females into non-diapause females induced diapause in 60% of the recipients. The above results were all significantly different from appropriate control injections. However, arguing from the incomplete termination of diapause in the experiment above and other results, the experimenters postulate a bifactorial control of diapause, wherein the gonadotropin is antagonized by a diapause-inducing factor. These factors remain to be isolated.

Interestingly, another population of *A. arboreus* that also parasitizes cattle egrets at the other end of the African continent in South Africa apparently also has a reproductive diapause, but at the opposite time of year, as might be expected at that latitude [117].

However, regardless of environmental conditions, most tick species are vulnerable to the intermittent availability of hosts, and so often have extended reproductive lapses with a concomitant need to conserve stored energy resources. With their ability to take many quick meals often in more protected nidicolous situations, soft ticks may appear less vulnerable to these lapses. However, having less mobility than non-nidicolous hard ticks, they are apparently even more at risk, reflected in females’ extraordinary ability to sustain themselves without food (*A. lahorensis*, 18 years, Pospelova-Shtrom (p. 72, [94]); *Ornithodoros moubata*, 5 years, [118]; *O tholozani*, 10 years, (p. 213, [94]); *A.* (*O.*) *brumpti*, 12 years, (volume 2, pp. 90–91, [119]) and in their ability to store sperm (see above). These abilities suggest specially adapted physiological mechanisms to maximize the retention of nutrients by minimizing metabolism and water loss. Ticks are well-known for their resilience to water loss and unusual ability to absorb water from low humidity environments, but other adaptations of their metabolism seem a fertile field for exploration.

## 13. Maternal Care

Ticks are commonly thought to lay large batches of eggs and then abandon them to fate. However, quite a few species among soft ticks have been shown to brood eggs and carry larvae for as much as many weeks. Female *A. arboreus* [53] brood eggs until hatching, *O. moubata* [91] and *Antricola marginatus* from Mexican bat caves [120] brood eggs and carry larvae. *Argas transgariepinus*, a widespread parasite of bats, and *Argas striatus*, a South African parasite of social weaver birds, brood eggs until hatching and can then carry larvae on the ventral side of their abdomens [121]. Female *A. transgariepinus* have unusual bowed setae on their abdomens and larvae have long claws and a pulvillus, both of which obviously enhance attachment. Several of the above authors and Hoogstraal (p. 155, [122]) have suggested that carrying larvae might be a way to facilitate getting the most vulnerable life stage of ticks to its first blood meal, but that has not yet been observed. Other possible functions might be predator protection or the reduction of water loss through aggregation.

## 14. Autogeny and Parthenogenesis

Autogeny (oviposition without a bloodmeal) is widespread but irregular in argasids. Two genera of argasids, *Antricola* and *Otobius*, are obligately autogenous; adults have reduced mouthparts that do not function in blood-feeding. *Antricola* do all their feeding as larvae [123], though *Otobius* feeds as larvae and nymphs. Many other argasids are facultatively autogenous: in an investigation of autogeny in nine *Ornithodoros* species, Feldman-Muhsam [124] found autogeny in three species but not in the other six species. However, she noted that autogenous oviposition was dependent on pre-adult ecological conditions, and Pound et al. [125] found that the expression of autogeny in *O. parkeri* was dependent on temperature. Facultative autogeny likely happens mostly in the absence of a ready blood meal, and typically only supports one gonotrophic cycle. The extraordinary *A. lahorensis*, a parasite of eastern Asian hoofed mammals, has an obligately autogenous first gonotrophic cycle, perhaps related to its unusual (among argasids) two-host lifestyle.

Parthenogenesis has been reported in several argasid species, but it is rare and has never been reported to produce viable adults. An experiment by Wagner-Jevseenko [13] tested 108 fed virgin female *O. moubata*: 24 oviposited, 9 developed larvae in the eggs, but none hatched.

## 15. Sexual Transmission of Parasites

The transgenerational (“vertical”) transmission of parasites in ticks happens primarily transovarially, but potentially can also happen venereally, through sperm or accompanying secretions passed to the female at mating. The transovarial transmission of viral, bacterial and protist parasites is widespread and has been discussed in several seminal reviews: Burgdorfer and Varma [126], Hoogstraal [122] and several chapters in Sonenshine and Roe [6], volume 2. As might be expected, the efficacy of transovarial transmission varies widely with the parasite species and host species, but it also apparently varies widely within a specific parasite–host relationship. For example, the importance of the transovarial transmission of the African relapsing fever spirochaete, *Borrelia duttoni*, to humans by *O. moubata* has been argued about for over half a century. The work of Aeschlimann and his associates documented ovarian infection and transmission (pp. 363–364, [127]), but the transmission of the parasites was found to be so rare in another study that its importance for disease transmission in comparison to lateral transmission between humans and ticks was considered to be insignificant [128]. As another example of the complexity of these relationships, the West Nile virus is passed transovarially in *A. arboreus* (but not in sympatric *A. persicus*) from adult female to larva, but it then does not survive trans-stadially, i.e., past the larval to nymph molt or the nymph to adult molt [129]. Another route for transmission, in this case the transmission of *Borrelia hermsi* by *Ornithodoros hermsi* [130], is by the phenomenon known as hyperparasitism or homovampirism, i.e., ticks feeding on other ticks, a phenomenon that has been reported in several other species of soft ticks (pp. 74–75, [94]).

The venereal transmission of parasites has often been cited as a possibility, but it has yet to be shown of importance. Working with *O. moubata*, Wagner-Jevseenko mated 107 male *O. moubata* infected with *B. duttoni* to uninfected females; only two of the females subsequently showed any infection with the spirochete, which the author considered epidemiologically insignificant [13]. Further, no venereal transmission was found in experiments by Abbassy et al. [129] where they mated 25 males infected with the West Nile virus to clean females.

**Figure 12:**
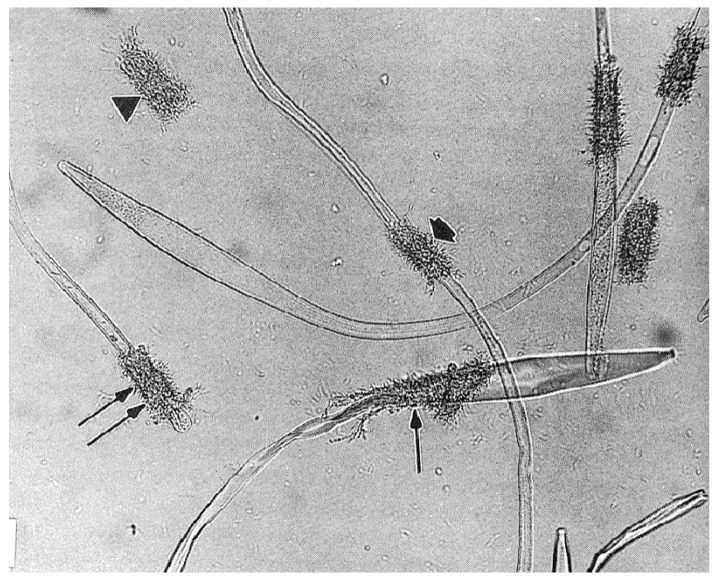
Adlerocystis symbionts on *Ornithodoros tholozani* spermatozoa. The original location is behind the head, as shown on the spermatozoon with the single long arrow. The remaining bunches of symbionts are in various stages of being shed as aggregates sliding posteriorly (other arrows). Published as: The role of *Adlerocystis* sp. in the reproduction of argasid ticks, B. Feldman-Muhsam, pp. 179–192. In: *The Acari: Reproduction*, *Development*, *and Life-history Strategies*, Schuster and Murphy, Eds., Chapman & Hall, London, UK, 1991. Copyright, Elsevier [133].

An interesting visible venereal transmission of symbiotes (not documented to be parasites) was discovered by Feldman-Muhsam and associates: a unicellular phycomycete fungus that they named *Adlerocystis* sp. that attaches to spermatozoa of certain argasid species [131]. The *Adlerocystis* are first found in the posterior lobes of the male’s accessory glands, where they appear as round, vacuolated cells. Upon mating, the cells are included in the ejaculated spermatophore, and after 4 h in the uterus begin multiplying and attaching to the developing spermatozoa behind the clavate head (Figure 12). Over the next 2 days, they continue to attach and transform into spindle-shaped cells. Electron micrographs show them attached but not penetrating the sperm membrane between the cellular processes. Perhaps significantly, some of the cellular processes of the spermatozoon send out processes to the *Adlerocystis* cells, and some *Adlerocystis* cells are included in the acrosomal canal. When the spermatozoa move out of the uterus, they slide out of the *Adlerocystis* as a unit, leaving the latter behind as a sort of sleeve, with the exception of those enclosed in the acrosomal canal [132]. This describes their fascinating trajectory in *O. tholozani* and *O. parkeri*, but in *O. moubata* and *A. persicus* they are found only as rounded cells that are included in the spermatophore but do not attach to the sperm [133]. The authors did not mention the further history of the cells, or what if any impact they might have on the female. Apparently, the taxonomic validity of *Adlerocystis* is in question [134].

## 16. Future Questions

1. Numerous jumping-off points have been elaborated above, but the following seem particularly salient and interesting:
2. Morphological studies have described several glands: in the male, the different functions of the complexly compartmentalized accessory glands beg definition, and in the female, possible secretory functions of the oviducts and the function(s) of the obvious secretions of the tubular accessory glands need elucidation.
3. Evidence that a carbon dioxide reaction blows the spermatophore of the male into the female has been provided by Feldman-Muhsam and associates [24], but the mechanism of its production needs further definition.
4. The mechanism and function of the many complex steps in the maturation of the unique tick spermatozoon need exploration.
5. The question of how tick spermatozoa fertilize eggs has received attention but no definitive solution for more than 100 years. It seems a carefully timed ultrastructural study might work.
6. The gliding motility of ticks and related mites is apparently unique and its mechanism would be a novel subject for a molecular cell biologist.
7. The stimulus that spermatozoa provide the female, as first identified by Aeschlimann [64], needs a molecular interpretation. Moreover, the resulting neurosecretory response would probably receive help from a transcriptomic analysis of soft tick synganglial secretion.
8. For oogenesis, vitellogenin secretion has received considerable attention, but we know little about the subsequent step of uptake into oocytes.
9. The two-phase regulation of oocyte maturation proposed by Horigane et al. [79] needs validation by documentation of how the food-induced pathway results in the only partial maturation of oocytes pending the full maturation induced by the mating pathway.
10. The scant evidence for volatile sex attractants in soft ticks points to a need for further substantiation and chemical analysis, as the identification of such pheromones in hard ticks has led to successful tick control measures [96].
11. The investigation of metabolic shutdown mechanisms in such long-lived organisms as soft ticks might have wider implications for all organisms.
12. The fate in the female of Feldman-Muhsam and Havivi’s symbiotes begs attention [131].
13. Many interesting questions of more behavioral ecological and evolutionary foci need attention, such as sexual selection mechanisms, the benefits/costs of few large vs. many small spermatozoa, the evolution of the gliding and vacuolated sperm of anactinotrichid (parasitiform) Acari [33].

In general, the advent of “omics” will of course transform our understanding of many of these issues, but also more traditional cell biological, biochemical, physiological and behavioral analyses that have been partially eclipsed by the “omics” need to be resuscitated. Amen.

## Data Availability

Not applicable.

## Supplementary Materials

The following supporting information can be downloaded at: https://www.mdpi.com/article/10.3390/pathogens12040582/s1, S1: Video of gliding motility of *Ornthodoros moubata* spermatozoa. S2: Video of head-curling of *Ornthodoros moubata* spermatozoa.
